# Mfn2 is Required for Mitochondrial Development and Synapse Formation in Human Induced Pluripotent Stem Cells/hiPSC Derived Cortical Neurons

**DOI:** 10.1038/srep31462

**Published:** 2016-08-18

**Authors:** Du Fang, Shijun Yan, Qing Yu, Doris Chen, Shirley ShiDu Yan

**Affiliations:** 1 Department of Pharmacology and Toxicology, and Higuchi Bioscience Center, School of Pharmacology, University of Kansas, Lawrence, KS, USA; 2 State Key Laboratory of Oral Diseases, West China Hospital of Stomatology, Sichuan University, Cheng Du, China

## Abstract

Mitochondria are essential dynamic organelles for energy production. Mitochondria dynamically change their shapes tightly coupled to fission and fusion. Imbalance of fission and fusion can cause deficits in mitochondrial respiration, morphology and motility. Mfn2 (mitofusin 2), a mitochondrial membrane protein that participates in mitochondrial fusion in mammalian cells, contributes to the maintenance and operation of the mitochondrial network. Due to lack of applicable model systems, the mechanisms and involvement of mitochondria in neurogenesis in human brain cells have not been well explored. Here, by employing the human induced pluripotent stem cells (hiPSCs) differentiation system, we fully characterized mitochondrial development, neurogenesis and synapse formation in hiPSCs-derived cortical neurons. Differentiation of hiPSCs to cortical neurons with extended period demonstrates mature neurophysiology characterization and functional synaptic network formation. Mitochondrial respiration, morphology and motility in the differentiated neurons also exhibit pronounced development during differentiation. Mfn2 knock-down results in deficits in mitochondrial metabolism and network, neurogenesis and synapse formation, while Mfn2 overexpression enhances mitochondrial bioenergetics and functions, and promotes the differentiation and maturation of neurons. Together, our data indicate that Mfn2 is essential for human mitochondrial development in neuronal maturation and differentiation, which will enhance our understanding of the role of Mfn2 in neurogenesis.

The continuous balance between fusion and fission is crucial for the quality control of mitochondrial metabolism and formation of a functionally network. The dynamin-related protein Drp1 mediates the mitochondrial fission and is involved in synapse formation and embryonic development^1^. Mitofusins (Mfn1 and Mfn2) are outer membrane GTPases that mediate outer mitochondrial membrane fusion. Mfn2 expression is crucial to the maintenance of the morphology and operation of the mitochondrial network and mitochondrial metabolism^2^. Mfn2 gene is located on chromosome 1p37 and its missense mutation, R104W at the critical GTPase domain has been associated with hereditary motor and sensory neuropathy, optic neuropathy, sensorineural hearing loss, and metabolic defects in occipital cortex^3^. Recent studies demonstrate that markedly reduced mitochondrial mass and transport may contribute to the neuronal loss due to specific loss of Mfn2 but not Mfn1^4^,^5^. Clinically, perturbations in mitochondrial dynamics have also been linked to several neurodegenerative diseases, including Alzheimer disease (AD) and Parkinson disease (PD)^5^,^6^,^7^,^8^.

Accumulating evidence has indicated the essential role of mitochondrial biogenesis in stem cell differentiation^9^,^10^,^11^,^12^. Mitochondrial and metabolic changes are regarded as hallmarks of differentiation processes in stem cells^12^. The ultrastructure and morphology of mitochondria undergo opposite and reversible changes during stem cells differentiation and reprogramming process. Elongation of mitochondrial network, whereas opposite remodeling of the mitochondrial network known as less mature mitochondrial, were observed in the processes of differentiation of human embryonic stem cells (hESCs) and reprogramming of human and mouse somatic cells into iPSCs, respectively^13^,^14^. Besides morphological and ultrastructural changes, increases in mitochondrial mass and mitochondrial DNA (mtDNA) levels and increased oxygen consumptions and reactive oxygen species (ROS) levels were observed during differentiation of hESCs, mESCs (mouse embryonic stem cells), and iPSCs (induced pluripotent stem cells)^10^,^15^,^16^. In addition, a metabolic transition from glycolysis to mitochondrial respiration was observed in both cardiac and motor neuronal directional differentiation^17^. Compared to hiPSCs, differentiated somatic cells display higher oxygen consumption rates, increased respiratory reserve capacity while decreased glycolysis with reduced lactate production^13^. Inhibition of mitochondrial respiration leads to the impairment of differentiation ability and enhancement of stem cell pluripotency^18^.

Overproduction of mitochondrial reactive oxygen species (mROS) has adverse effects on cellular function including iPSCs^19^,^20^,^21^,^22^. In iPSCs-induced neurons derived from AD and PD patients, mitochondrial dysfunction and abnormally high levels of ROS result in increased vulnerability of these cells^6^,^23^,^24^. However, mROS are also involved in cellular processes that are critical for maintenance of homeostasis and adaptation to stress^25^. ROS are involved in the regulation of proliferation and differentiation of neural progenitor cell (NPCs). Rapid bursts of superoxide radical anions called superoxide flashes, could regulate the self-renewal and differentiation of mouse embryonic NPCs^26^. Therefore, low levels of ROS are necessary to preserve the stemness, whereas increased ROS levels favor differentiation.

Mechanisms underlying mitochondrial defects in hiPSC-derived neurons are not well understood. In the present study, we addressed the role of Mfn2 in the hiPSCs differentiation system and report here that knockdown of Mfn2 results in mitochondrial dysfunctions and defects in neurogenesis and synapse formation. On the contrary, Mfn2 overexpression of neural progenitor cells (NPCs) directs differentiation and maturation into neurons with enhanced mitochondrial functions. These data indicate that Mfn2 is crucial to mitochondrial development, and thereby essential to hiPSCs differentiation.

## Results

### Cortical neuron-directed hiPSC differentiation

We first assessed pluripotency in BM2-3 hiPSCs. These cells were immunoreactive for pluripotency markers: Nanog (Supplementary Figure S1AI), OCT4 (Supplementary Figure S1AII), SSEA3 (Supplementary Figure S1AV) and SOX2 (Supplementary Figure S1AVI). Our iPSCs were successfully kept in an undifferentiated state for more than 17 passages. The cells (17 passages) formed uniform-sized embryoid bodies (EBs) 24 hours after being plated into AggreWell™800 plates (Supplementary Figure S1B). 1–2 days after attachment, prominent neural rosette structures appeared in neural aggregates. About 98% of attached neural aggregates had polarized rosettes covering >50% of their area (Supplementary Figure S1C). Rosettes were formed by cells expressing proteins characteristic of progenitor markers of PAX6 (Supplementary Figure S1C).

**Figure 1:**
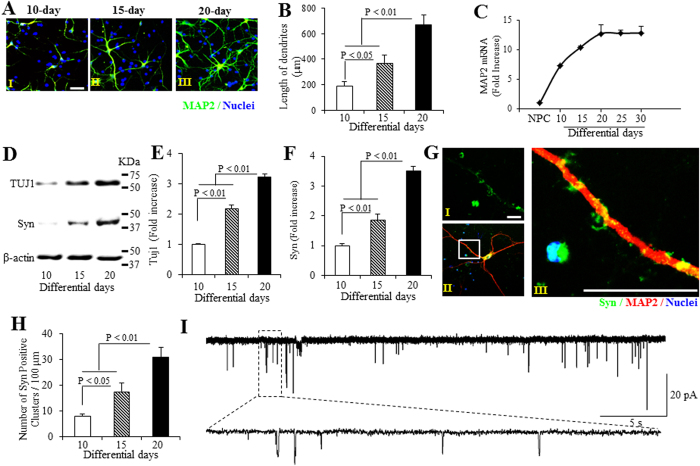
Differentiation and synaptic maturation of hiPSC line-induced human cortical neurons. (**A–C**) Assessment of neuronal process length and neuronal marker microtubule-associated protein 2 (MAP2) mRNA level in the hiPSC line-induced human cortical neurons differentiated for different days. (**A**) Representative images for immunostaining of MAP2 (green, **A**) of human cortical neurons underwent 10- (AI), 15- (AII) and 20-day (AIII) differentiation. (**B**) Quantification of neuronal process length in MAP2-positive neurons, and **(C)** Quantitative real-time PCR for analysis of MAP2 mRNA level differentiated for 10 to 30 days. (**D–F**) Analysis of immunoblots for neuron-specific marker (Neuron-specific class III beta-tubulin, TUJ1) and synaptophysin (Syn) protein expression. (**D**) Representative immunoblots for TUJ1, Syn, and β-actin. Quantifications of TUJ1 (**E**) and Syn (**F**) expression levels are normalized to β-actin using NIH image J program (n = 3). (**G**) Representative images for immunostaining of Syn (green, GI), co-immunostaining of Syn (green) and MAP2 (red, GII), and (GIII), enlargement of (GII) of human cortical neurons underwent 20-day differentiation. (**H**) Number of Syn-positive clusters along with the branches of hiPSC-derived cortical neurons cultured in differentiation media for different days was performed using NIH Image J program. **(I)** Representative spontaneous postsynaptic currents (sPSCs) recorded from hiPSC-derived cortical neurons cultured in differentiation media for 20 days. Scale bars = 20 μm in (**A**,**G)**.

The neuronal marker microtubule-associated protein 2 (MAP2) was significantly increased over time (Fig. 1A,B). We observed significant growth of dendrites, as shown by measurement of neuronal process length during the course of differentiation, with the longest processes noted on day-20 (Fig. 1A,B). MAP2 mRNA levels were significantly increased over differentiated time (Fig. 1C). Immunoblotting of cell lysates demonstrated that that expression levels of neuron-specific markers [synaptophysin (Syn) and neuron-specific class III beta-tubulin (TUJ1)] were significantly increased from day 10 to day 20 neurons, and reached plateau after day-20 differentiated neurons (Fig. 1D–F, Supplementary Figure S2A–C). These data indicate that neurons can be induced to the mature neuronal characteristics in our differentiation system.

Synaptogenesis is a critical indicator in neural network formation. Using immunostaining, we demonstrated that the number of pre-synaptic compartment (syn-positive puncta) were increased with neuronal differentiation for 10, 15 and 20 days (Fig. 1G,H), which is consistent with immunoblotting results showing that synaptophysin expression levels were elevated over time of induction, and reached its highest level 20 days after differentiation (Fig. 1F–H, Supplementary Figure S2A,C).

To investigate the formation of functional excitatory synapses in hiPSCs differentiated cortical neurons, we next performed a whole-cell patch-clamp recording to detect spontaneous postsynaptic currents (sPSCs) in day-20 neurons to assess their neuronal function. As shown in Figure 1I, fourteen cells (5.8%, n = 242) exhibited appearance of sPSCs, suggesting that the differentiated cortical neurons have the capacity to form functional synapses. These data demonstrate that functional and excitatory synapses are generated during the course of differentiation.

### Physiological characteristics of differentiated neurons

**Figure 2:**
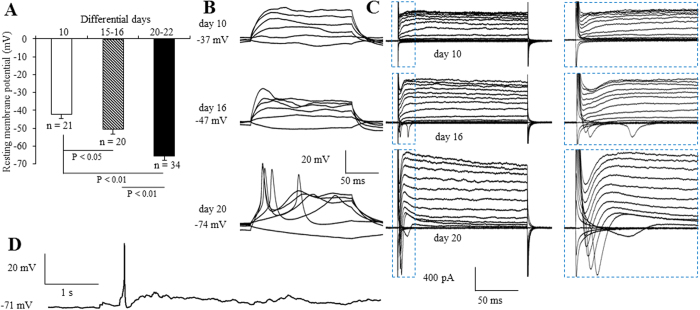
Electrophysiologic characterization of hiPSC-derived neurons. (**A**) Quantification of resting membrane potential in hiPSC-derived cortical neurons cultured in differentiation media for 10, 15~16 and 20~22 days. (**B**) Representative traces of membrane potential responding to step depolarization by current injections ranging from −10 pA to 90 pA at 20 pA increments. Membrane potential was current-clamped at the resting membrane potential. (**C**) Representative traces of whole-cell currents in voltage-clamp mode; cells were held at −70 mV; step depolarization from −90 mV to +50 mV at 10-mV intervals was delivered. The inset shows Na^+^ currents. (**D**) Spontaneous action potentials recorded from a cortical neuron 20 days after differentiation. No current injection was applied.

To further determine whether cortical neurons derived from hiPSCs actually have neurophysiologic function, we next examined the electrophysiological properties of hiPSC-induced neurons. During differentiation, the membrane was maturated over time as shown by the resting membrane potential (Fig. 2A). As further evidence of maturation, the ability to fire bursts of action potentials in response to current injection increased over time (Fig. 2B). None of the neurons exhibited action potentials until day 10 of the incubation in differential media. We observed a single action potential appeared until day 16 (Fig. 2B). The frequency continued to increase up to day 20 (Fig. 2B). Electrophysiological parameters such as resting membrane potential and action potential generation showed signs of membrane maturation over time (Fig. 2A,B). In voltage-clamp mode, we observed fast inactivating inward and outward currents, which probably correspond to the opening of voltage dependent Na^+^ and K^+^ channels, respectively (Fig. 2C). In addition, 4 cells (1.7%, n = 242) showed spontaneous action potentials, some as early as 16 days after transduction (Fig. 2D). Taken together, these data demonstrate that hiPSC-derived neurons are functional after 16 days of differentiation in our neuronal induction system.

### Alteration of mitochondrial functions in hiPSCs differentiated cortical neurons

**Figure 3:**
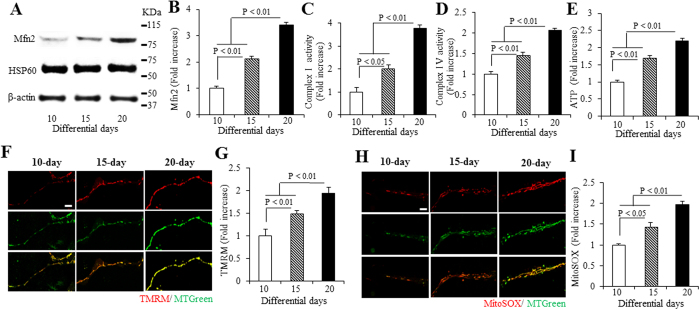
Mfn2 expression, mitochondrial functions, and ROS in hiPSC-derived cortical neurons. (**A,B**) Immunoblotting for Mfn2 expression in hiPSC-derived neurons differentiated for 10, 15 and 20 days. (**A**) Representative immunoblots for Mfn2, Hsp60, and β-actin. (**B**) Quantification of Mfn2 expression level is normalized to β-actin using NIH image J program (n = 3). (**C–E**) Enzymatic activity of complex I (**C**), IV (**D**), and cellular ATP levels (**E**) were determined in cell lysates from hiPSC-derived cortical neurons cultured in differentiation media for 10, 15 and 20 days. Data are expressed as fold increase relative to the neurons cultured in differentiation media for 10 days. Mitochondrial membrane potential and ROS levels were evaluated by TMRM (**F**,**G**) and MitoSOX staining (**H**,**I**), respectively. (**F**,**H**) Representative images for TMRM (**F**) and MitoSOX (**H**) staining for hiPSC-derived neurons cultured in differentiation media for 10, 15 and 20 days. Quantifications of immunofluorescent intensity for TMRM and MitoSOX are shown in (**G**,**I)**. Scale bars = 5 μm in (**F**,**H**).

We first investigated the level of Mfn2 in cortical neurons derived from hiPSCs for 10 to 20 days. Mfn2 levels increased with days of differentiation. There are a 2.1 and 3.4 folds increases in day 15 and 20 compared to day 10, respectively (Fig. 3A,B). It was noted that Mfn2 expression level reached its highest level 20 days after differentiation (Supplementary Figure S2A,D). Given the important role of mitochondria in neuronal transmission and function, we evaluated mitochondrial respiratory function and energy metabolism by measuring enzymatic activity for complexes I and IV, key enzymes associated with the respiratory chain, and ATP levels in hiPSC-derived cortical neurons. Complex I and IV activity was significantly increased on day 15 and 20, compared to day 10 differentiated neurons (Fig. 3C,D). Similarly, ATP levels were significantly elevated on day 15 to 20 compared to those from day 10 (Fig. 3E). These results suggest enhanced mitochondrial respiratory function and energy metabolism during the course of maturation of hiPSC- induced cortical neurons.

Overproduction of mitochondria ROS interferes with cellular function, thereby accelerating pathologies and aging^23^. In contrast, recent work has revealed that mitochondrial ROS are integral signaling molecules that promote proliferation, and possibly also control differentiation^20^,^21^,^25^. We therefore assessed mitochondrial ROS levels with MitoSOX staining and association of ROS with mitochondrial membrane potential using tetraethylrhodamine methyl ester (TMRM) staining in the neuronal terminals upon differentiation. Mitochondrial membrane potential was significantly increased by 1.5- and 1.9-fold in day 15 and 20 neurons, respectively (Fig. 3F,G). Accordingly, in day 15 and 20 of differentiated neurons, the intensity of MitoSOX staining was increased by 1.4- and 2.0-fold (Fig. 3H,I). Similarly, using highly sensitive and specific electron paramagnetic resonance (EPR) spectroscopy to evaluate intracellular ROS levels, ROS levels were increased by 1.7- and 2.1-fold (Supplementary Figure S3A,B). The membrane potential and mROS reached their highest levels in day-20 neurons (Fig. 3F–I). These data suggest that ROS levels were positively correlated with the progression of hiPSC cell development.

### Knockdown of Mfn2 inhibits mitochondrial functions in hiPSCs differentiated neurons

**Figure 4:**
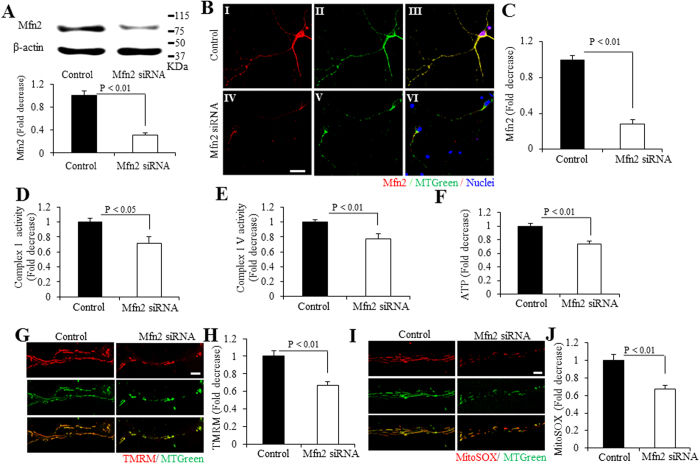
Effect of Mfn2 knockdown on the development of mitochondrial functions in hiPSC-derived neurons. (**A–C**) Analysis of immunoblotting and immunostaining for Mfn2 expression in hiPSC-derived neurons differentiated for 20 days with Mfn2 siRNA treatment. (**A**) Quantification of Mfn2 expression level is normalized to β-actin using NIH image J program (n = 3). Representative immunoblots for Mfn2 expression and β-actin are shown in the upper panel of (**A)**. (**B**) Representative images for immunostaining of Mfn2 (Red, BI and IV), MTGreen (green, a marker for mitochondria, green, BII and V) and merge (yellow, BIII and VI) of human cortical neurons underwent 20-day differentiation, and quantification of immunostaining density is shown in (**C)**. Scale bars = 15 μm in (**B)**. (**D–F**) Enzymatic activity of complex I (**D**), IV (**E**), and cellular ATP levels (**F**) were determined in cell lysates from hiPSC-derived cortical neurons cultured in differentiation media for 20 days, with Mfn2 siRNA or control siRNA treatment. Data are expressed as fold increase relative to the neurons cultured in differentiation media for 20 days without Mfn2 siRNA treatment. Mitochondrial membrane potential and ROS levels were measured by TMRM (**G,H)** and MitoSOX staining (**I,J**), respectively. Representative images for TMRM (**G**) and MitoSOX (**I**) staining for hiPSC-derived neurons cultured in differentiation media for 20 days. Scale bars = 5 μm in (**G**,**I)**. Quantifications of immunofluorescent intensity for TMRM and MitoSOX are shown in (**H**,**J)**, respectively.

To determine the effect of Mfn2 on neuronal differentiation, we knocked down Mfn2 in hiPSCs differentiated neurons. The cells were transfected with Mfn2 siRNAs for 8 days, started from day 12 after incubated in neural expansion medium. In day 20, Mfn2 expression level was reduced by ~70% in hiPSCs differentiated neurons treated with siRNA-Mfn2 by immunoblot of cell lysates for Mfn2 (Fig. 4A). Consistent with immunoblotting results, Mfn2 staining intensity was significantly decreased by ~70% (Fig. 4B,C). With Mfn2 knockdown, activities for complexes I and IV, and ATP levels in hiPSC-derived cortical neurons in day 20 were all significantly reduced (Fig. 4D–F). Similarly, knockdown of Mfn2 expression reduced the levels of TMRM and MitoSOX staining intensity (Fig. 4G–J), and also decreased the intracellular ROS levels disclosed by our EPR results (Supplementary Figure S3C,D). These results indicate that Mfn2 is required in the mitochondrial functions and maturation in hiPSCs differentiated cortical neurons.

### Knockdown of Mfn2 inhibits the differentiation and synaptogenesis of hiPSC -induced neurons

**Figure 5:**
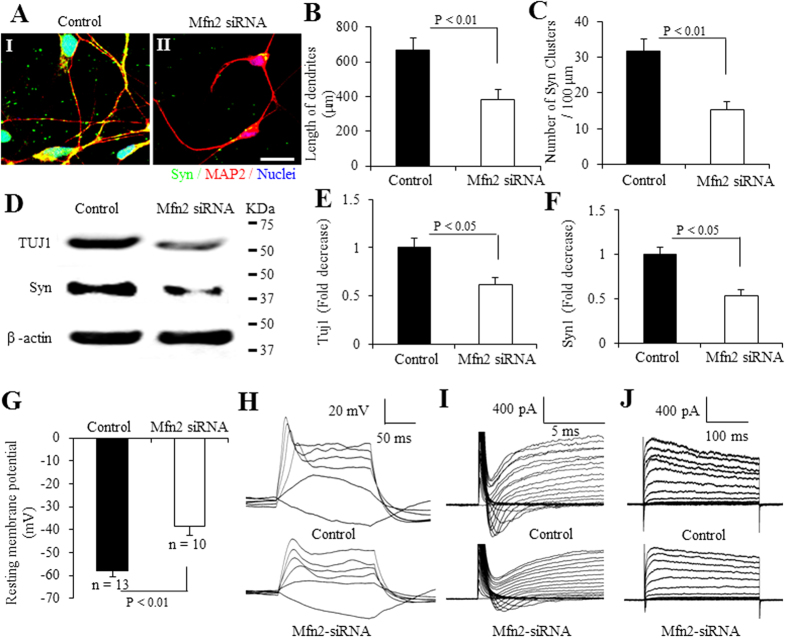
Involvement of Mfn2 in differentiation and synaptic maturation of hiPSC-induced neurons. (**A–C**) Assessment of neuronal process length and number of synaptophysin (Syn)-positive clusters along the branches of hiPSC-derived cortical neurons with Mfn2 siRNA or control siRNA treatment. Representative images for immunostaining of MAP2 (red, **A**) and synaptophysin (Syn) (green, **A**) of human cortical neurons underwent 20-day differentiation with Mfn2 siRNA (AII) or control siRNA treatment (AI). Scale bars = 20 μm. Quantification of neuronal process length of MAP2-positive neurons (**B**) and number of Syn-positive clusters along the branches of hiPSC-derived cortical neurons **(C)** cultured in differentiation media for 20 days with Mfn2 siRNA or control siRNA treatment, using NIH Image J program. (**D–F**) Quantifications of TUJ1 (**E**) and Syn (**F**) expression levels are normalized to β-actin using NIH image J program. Representative immunoblots are for the indicated proteins (**D**). Resting membrane potential in hiPSC-derived neurons with control siRNA or Mfn2 siRNA transfection are calculated **(G**). (**H**) Representative traces of action potentials elicited from hiPSC-derived neurons with control siRNA (up image) or Mfn2 siRNA transfection (below image). Cells were maintained at the resting membrane potential. Step current injection protocols were used from −10 to 90 pA at 20 pA increments. (**I,J**) Representative traces of Na^+^ currents (inward) and K^+^ currents (outward) in hiPSC-derived neurons with indicated treatments are shown in (**I**,**J)**, respectively. Cells were clamped at −70 mV. Step depolarizations from −70 to +50 mV at 10 mV increments were delivered.

Next, we investigated the ability of differentiation with Mfn2 knockdown. The length of neuronal dendrites indicated by MAP2 immunostaining (Fig. 5A,B) and TUJ1 expression level (Fig. 5D,E) was significantly decreased with Mfn2 knockdown. To synaptogenesis, Mfn2 knockdown also decreased the number of Syn-positive puncta (Fig. 5A,C) and Syn protein expression level (Fig. 5D,F) in day 20 neurons. Similarly, hiPSC-derived neurons with Mfn2-siRNA transduction showed lower negative resting membrane potential (Fig. 5G). The action potential was significantly lower in Mfn2-siRNA-treated neurons than control siRNA-treated neurons, suggesting reduced ability of eliciting action potentials in Mfn2 knockdown neurons. (Fig. 5H). The membrane Na^+^ and K^+^ channels also appeared to be immature (Fig. 5I,J). These data indicate that Mfn2 is essential for the differentiation and synaptogenesis of hiPSC-induced neurons.

### Knockdown of Mfn2 alters mitochondrial morphology and mobility in hiPSC differentiated neurons

**Figure 6:**
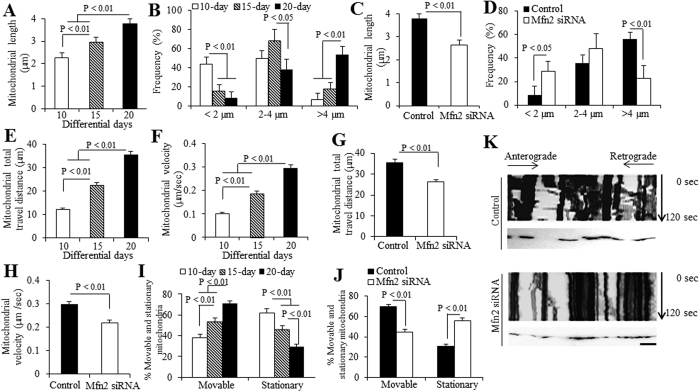
Effect of Mfn2 on the development of mitochondrial morphological and dynamic parameters in hiPSC-derived neurons. In neurons differentiated for varying days, average (**A**) and frequency (**B**) of mitochondrial length in the processes of neurons were quantified. Effect of Mfn2 siRNA treatment on morphological changes [(**C)** for average mitochondrial length and (**D)** for frequency] were also quantified. Average mitochondrial travel distance (**E**) and mitochondrial travel velocity (**F**) were calculated in the processes of neurons differentiated for varying days. Effect of Mfn2 siRNA treatment on mitochondrial dynamic parameters [(**G)** for mitochondrial travel distance and (**H)** for travel velocity] were also measured. The percentage of stationary and moveable mitochondria was compared in hiPSC-derived neurons differentiated for varying days (**I**), and with the effect of Mfn2 siRNA treatment (**J**). (**K**) Kymographs generated from live imaging movies represent hiPSC-derived neurons cultured in differentiation media for 20 days, with or without Mfn2 siRNA treatment. In the kymographs, the X-axis is mitochondrial position and the Y-axis represents the time lapse of 0–120s. Vertical white lines represent stationary mitochondria and diagonal lines represent moving mitochondria. Anterograde movements are from left to right and retrograde movements are from right to left. Scale bar = 10 μm in (**K)**.

We then investigated the effect of Mfn2 in the development of mitochondrial morphology and mobility in the processes of hiPSC differentiation to cortical neurons. Quantification of MTGreen-positive mitochondria, we observed that mitochondria on 10, 15 and 20 day neurons became longer with differential days (Fig. 6A). The average length of mitochondria was 2.27 μm, 2.96 μm and 3.78 μm of mitochondria on day 10, 15 and 20, respectively (Fig. 6A). Most mitochondria were shorter than 2 μm or ranged from 2–4 μm on day 10, while most mitochondria in day 20 were longer than 4 μm (Fig. 6B). The elongated mitochondrial shape and increased mitochondrial content (MTGreen staining in Fig. 3) could be the results from upregulated Mfn2 level with differentiation overtime (Fig. 3A,B). With Mfn2 knockdown in 20-day neurons, the average length of mitochondria was significantly reduced in 20-day neurons compared to those treated with control siRNA (Fig. 6C), and most mitochondria were in the range of 2–4 μm (Fig. 6D), which confirmed the role of Mfn2 in regulation of mitochondrial morphology and content.

To mitochondrial mobility, we investigated dynamic indicators of mitochondria movement. Compared to the mitochondria from the day 10 differentiated neurons, mitochondria displayed longer travel distance (Fig. 6E) with significantly higher movement speed (Fig. 6F) in neuronal processes of hiPSC-derived neurons differentiated for 15 and 20 days. Mfn2 knockdown significantly decreased both travel distance (Fig. 6G) and movement speed (Fig. 6H) in day 20 differentiated neurons. The percentage of moveable mitochondria increased over time, while the percentage of stationary mitochondria decreased over the time course of differentiation and maturation (Fig. 6I). Mfn2 knockdown reduced the percentage of moveable mitochondria but increased the percentage of stationary mitochondria (Fig. 6J). Representative kymograph images showed less moveable mitochondria in the process of the day 20 differentiated cortical neuron with Mfn2 knockdown (Fig. 6K), as compared with those treated with control siRNA. These results demonstrate that knockdown of Mfn2 expression levels inhibit mitochondrial development of hiPSC-derived neurons during differentiation.

### Mfn2 overexpression promotes differentiation and synaptogenesis of hiPSC-induced neurons

**Figure 7:**
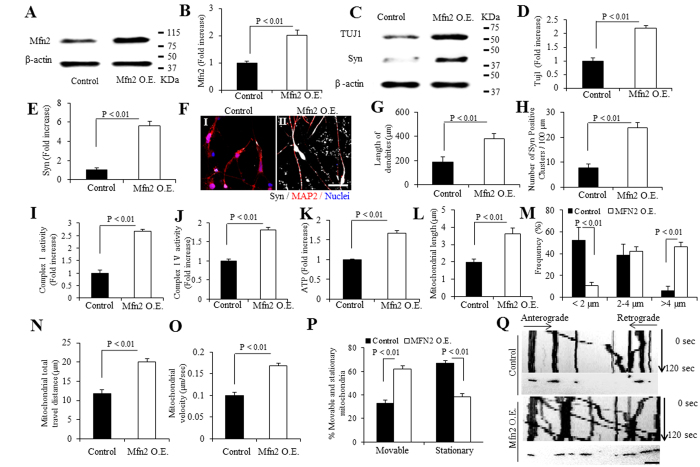
Effect of Mfn2 overexpression on the differentiation of hiPSC-derived neurons and development of mitochondrial functions. (**A,B**) Immunoblotting for Mfn2 expression in hiPSC-derived cortical neurons differentiated for 10 days with Mfn2 overexpression (O.E.) and representative immunoblots for Mfn2 expression and β-actin. (**B**) Quantification of Mfn2 expression level is normalized to β-actin using NIH image J program. (**C**) Immunoblotting for TUJ1 and Syn proteins expressions and representative immunoblots for TUJ1, synaptophysin (Syn) and β-actin. Quantifications of TUJ1 (**D**) and Syn (**E**) expression levels are normalized to β-actin. (**F–H**) Neuronal process length and number of Syn-positive clusters along the branches of hiPSC-derived cortical neurons with Mfn2 O.E. Representative immunostaining images for MAP2 (red, **F**) and Syn [(Grey, color changed from far red, **(F)**] of human cortical neurons underwent 10-day differentiation with Mfn2 O.E. (FII) or vehicle treatment (FI). Scale bar = 20 μm in (**F)**. Quantification of neuronal process length of MAP2-positive neurons (**G**) and number of Syn-positive clusters along the branches of hiPSC-derived cortical neurons **(H)** cultured in differentiation media for 10 days with Mfn2 O.E. or vehicle treatment. (**I–K**) Enzymatic activity of complex I (**I**), IV (**J**), and ATP levels (**K**) were determined in cell lysates from hiPSC-derived cortical neurons cultured in differentiation media for 10 days, with or without Mfn2 O.E. Data are expressed as fold increase relative to the neurons cultured in differentiation media for 10 days with vehicle treatment. **(L–Q)** Effect of Mfn2 O.E. on mitochondrial development [(**L)** for average mitochondrial length and (**M)** for frequency]. Average mitochondrial travel distance (**N**) and velocity (**O**) were calculated in the processes of neurons. The percentage of stationary and moveable mitochondria was compared (**P**). (**Q**) Kymographs generated from live imaging movies re present hiPSC-derived cortical neurons cultured in differentiation media for 10 days, with or without Mfn2 O.E. In the kymographs, the X-axis is mitochondrial position and the Y-axis represents the time lapse of 0–120 s. Vertical white lines represent stationary mitochondria and diagonal lines represent moving mitochondria. Anterograde movements are from left to right and retrograde movements are from right to left. Scale bar = 10 μm in (**Q)**.

We finally investigated the effect of Mfn2 overexpression on the differentiation and synaptogenesis in hiPSC-induced neurons. Cells were transfected with Mfn2 plasmid at NPCs stage. Mfn2 expression level was significantly elevated by ~2-fold compared to vector-transfected day 10 neurons by immunoblotting of cell lysates for Mfn2 (Fig. 7A,B). Synaptic proteins including TUJI and synaptophysin were increased in Mfn2-overexpressed neurons (Fig. 7C–E). Accordingly, Mfn2-overexpressed neurons displayed an increase in the length of dendrites as shown by MAP2 immunostaining (Red, Fig. 7F,G) and the number of synpatophysin (Syn)-positive puncta (Grey, color changed from far red; Fig. 7F,H). These results demonstrate a positive effect of Mfn2 on neuronal differentiation and synaptogenesis.

### Mfn2 overexpression enhances mitochondrial bioenergetics and functions

We next evaluated mitochondrial function by measuring activities for complexes I and IV, and ATP levels. These parameters were significantly increased in Mfn2 overexpressed neurons (Fig. 7I–K). Intriguingly, Mfn2 overexpression revealed an increase in intracellular ROS levels (Supplementary Figure S3E,F). These results indicate that Mfn2 is required in the mitochondrial functions and maturation in hiPSCs differentiated cortical neurons.

To mitochondrial morphology and mobility development, the average length of mitochondria was significantly increased in Mfn2-overexpressed 10-day neurons compared to those with vehicle treatment (Fig. 7L). The most mitochondria shifted to over 4 μm of length (Fig. 7M). Mitochondria from Mfn2 overexpressed neurons demonstrated to be more “active”, displaying longer travel distance (Fig. 7N), higher movement speed (Fig. 7O) and more moveable mitochondria (Fig. 7P,Q). Collectively, these results demonstrate that Mfn2 plays an active role in mitochondrial development of hiPSC-derived neurons during differentiation, which are required for neuronal differentiation and synaptogenesis.

## Discussion

Highly proliferative cells of iPSCs, prefer to undergo glycolysis and reduce ATP production by mitochondria, thus to maintain a low level of mitochondrial oxidative stress. Substantial mitochondrial structural and functional changes can disturb stem cell proliferation and differentiation. Mediated by the mitofusins (Mfn), dynamin-related protein 1 (Drp1) and dynamin-related GTPases, mitochondrial fusion and fission play an active role in regulating mitochondrial structural and functional changes. During differentiation, pluripotent cells show more polarized mitochondria, poised to respond to an increase in energy demand. Drp1 activation induces mitochondrial fragmentation, which favors to the acquisition and maintenance of stem cell pluripotency^27^. Although both Mfn1 and mitofusin 2 (Mfn2) mediate the fusion of mitochondrial membranes, malfunction of Mfn2, but not Mfn1 has been demonstrated to result in significant reduced mitochondrial transport, impaired respiratory chain function, and aberrant morphology in mitochondria, contributing to degeneration of dopaminergic neurons^4^,^5^. Mfn2 is necessary for embryonic development^28^. Here, we first observed that Mfn2 expression level was increased during differentiation. In our differentiation system, Mfn2 level reaches the maximum at day 20 in neuronal differentiation. The increase in Mfn2 expression levels leads to an elongated mitochondrial shape and increased mitochondrial content, indicating that Mfn2 is essential for regulation of mitochondrial morphology by tipping the balance in favor of mitochondrial fusion.

Compared to day 20 neurons, no difference in Mfn2 level was obtained in differentiated cells from day 30 (Supplementary Figure S2A,D) and 2-month (data not shown), suggesting the completed differentiation and maturation of hiPSCs to cortical neurons at day 20. We have provided the following evidence that day 20 differentiated functioning neuronal lineage cells are mature cortical neurons: 1) Immunoblotting of cell lysates demonstrated that expression levels of neuronal specific markers (TUJI, MAP2, and synaptophysin, a major synaptic vesicle protein) increased over time, plateauing after day 20 differentiated neurons; 2) Similar results were obtained from quantitative real-time PCR showing a time-dependent increase in neuronal marker MAP2 that reached to the plateau on day 20; 3) Immunostaining verified that day 20 differentiated neurons expressed neuronal markers MAP2 and synaptophysin with a typical neuronal morphology including a long neuronal process and dendrites. Lastly, neurophysiology data showed that the resting membrane potential reached −74 mV on day 20 differentiated neurons, which indicates maturation of cortical neurons as the membrane potential of neurons is lower than −60 mV.

We therefore started to knock down Mfn2 in day 12, before it reaches its maximum level. About a 70% reduction in Mfn2 was attained after 8 days incubation with Mfn2 siRNA treatment. With Mfn2 knock down, more fragmental mitochondria (<2 μm) were observed, accompanied by defects in mitochondrial function as less matured mitochondria. Accordingly, the reduced levels of Mfn2 significantly inhibited the mobility of mitochondria as shown by trends in lower mitochondrial moving speed and shorter travel distance in the differentiated processes. This could account for the lower ATP and complex activities with Mfn2 silence. These data demonstrate the effect of Mfn2 on mitochondrial functional and morphological development during hiPSCs differentiation, which correlates to the defects in mitochondrial respiratory function and ATP levels for the development.

Consistent with results showing the defects and malfunctions in mitochondria, Mfn2 silence also resulted in less maturation and synaptic mal-development of neurons under differentiation in this study. Length of neuronal processes and the density of synaptophysin puncta were significantly decreased in the day 20 neurons with Mfn2 siRNA treatment. Given that the differentiation of stem cells is vulnerable to ATP production and that mitochondria are regarded as major sites of cellular respiration and energy production^12^, Mfn2-mediated perturbation of mitochondrial fusion property and function may be responsible for the mal-development of differentiating and maturating iPSC-derived cortical neurons. The importance of Mfn2 involved in neuronal differentiation and mitochondrial development is confirmed in the Mfn2 overexpressed model of hiPSCs-direct neurons during differentiation. We further overexpressed Mfn2 from the neural progenitor cells (NPCs). As expected, after 10 days differentiation, neurons overexpressing Mfn2 exhibited more mature like phenotype, with more developed mitochondrial functions and higher mitochondrial membrane potential level. Overproduction of ROS is known to be involved in DNA damage, protein modification and lipid peroxidation of membranes. Growing body of recent evidence has demonstrated that ROS, in a certain level, are essential in abundant physiological and pathophysiological processes *via* covalent modifications to modulate various intracellular signaling pathways. Maintaining low level of ROS levels is necessary to preserve the proliferation of stem cells. Differentiating neurons and astrocytes displays up-regulated levels of ROS compared with their neuronal progenitors counterparts^29^. It is also suggested that the stimulating effect of ROS on cardiomyocyte differentiation^30^. Our study demonstrate increased levels of mROS as well as intracellular ROS (Supplementary Figure S3A,B) over the periods of differentiation, whereas Mfn2 knock down reduced ROS levels (Supplementary Figure S3C,D), in parallel, inhibited differentiation ability, reduced activities of enzymes associated with complex I and IV, and intensities of TMRM staining. On the contrary, overexpressing Mfn2 from NPCs stage, promoted neuronal differentiation ability, increased activities of enzymes and intensities of TMRM staining, along with elevated ROS levels (Supplementary Figure S3E,F). Our previous studies demonstrate that an increased expression level of Mfn2 positively correlates with oxidative stress in cybrid cells containing mild cognitive impairment (MCI)-derived mitochondria. Blockage of Mfn2 expression by genetic knockdown of Mfn2 significantly suppresses ROS production, attenuates aberrant mitochondrial morphology and function, and restores mitochondrial fission and fusion balance^7^. These data indicate that Mfn2-involved regulation of ROS contributes to neuronal differentiation process and mitochondrial maturation in addition to pathological events.

Taken together, using neurophysiologic model of mitochondrial development during neurogenesis of cerebral cortical neurons derived from hiPSCs, we provide new insight into the role of Mfn2 in the mitochondrial function, dynamics, and maturation, and properties of neuronal structure, synaptic formation during the course of formation and maturation of human cortical neurons. Knock-down Mfn2 results in imbalance of mitochondrial dynamics by diminishing mitochondrial fusion event, leading to mitochondrial function and network, neurogenesis and synapse formation, while overexpressing Mfn2 promotes the differentiation and maturation of neurons. Mfn2 has been reported to tether the endoplasmic reticulum to mitochondria^31^,^32^, the observed phenotype of impaired neuronal lineage differentiation with Mfn2 knockdown defects could potentially result from ER-mitochondria coupling, which requires further investigation. These results suggest the key factor of Mfn2 in mitochondrial dynamics and maturation, which are essential in human neuronal maturation and differentiation. Nevertheless, our studies also provide a novel neurophysiologic model of mitochondrial development in neurogenesis, which enhance our understanding the involvement of dysfunctional mitochondria in aging and neurodegenerative diseases. In view of protective effect of increased expression of Mfn2 on mild cognitive impairment (MCI)-impaired mitochondrial fusion/fission balance and mitochondrial alteration^7^, we propose that generation of Mfn2 agonists may hold potential for preventing or treating of neurodegenerative diseases including Alzheimer’s disease^8^ by augmenting mitochondrial function and neurogenesis.

## Methods

Generation and use of human iPSC was approved by University of Kansas on Human Studies. All methods were performed in accordance with the approved guidelines.

### hiPSC culture

Bone marrow 2–3 (BM2-3) from hiPSCs passaged 16–17 times (P16-17) (obtained from Dr. Sunita L. D’Souza, department of Gene and Cell Medicine and Black Family Stem Cell Institute, Mount Sinai School of Medicine, & department of Developmental and Regenerative Biology, Icahn School of Medicine at Mount Sinai, New York) were maintained under feeder-free conditions using matrigel (BD Biosciences)-coated 6-well tissue culture plates in Essential 8™ Medium (E8, Life technologies), supplemented with 10 μM ROCK inhibitor Y27632 (Life technologies) on passaging days. Cells were routinely passaged as small clumps using a previously described EDTA method^33^. We obtained informed consent from all IPS cells derived human subjects.

### Formation of embryoid bodies (EB) and induction of rosette

Feeder-free iPSCs were dissociated with TryplE and seeded onto AggreWell™800 plate (10,000 cells per EB; Stem Cell Technologies) in E8 media supplemented with 10 μM ROCK inhibitor Y27632 for the first 24 h; we changed 75% of the media daily with STEMdiff™ Neural Induction Medium (NIM, Stem Cell Technologies). EBs were harvested after 5 days and plated onto poly-L-ornithine/laminin (PLO/L, Sigma) coated plates. 1–2 day(s) after attachment, prominent neural rosette structures were visible inside the attached neural aggregates. Rosettes were formed by cells expressing proteins characteristic of progenitor marker of PAX6, as described in Supplementary Information.

### Isolation of stem/progenitor cells and differentiation of cortical neurons

We followed the differentiation protocol^34^ with some modifications. Briefly, stem/progenitor cells from neuronal rosette clusters were isolated using Neural Rosette Selection Reagent (Stem Cell Technologies) 5 days after incubation in NIM. Detached cells were collected and plated onto poly-L-ornithine and laminin (PLO/L) coated 6-well plates for Immunoblotting analysis and complex activities measurement, coverslips for immunocytochemistry and electrophysiology or chamber slides (Labtek) for mitochondria morphology and movement analysis. Neural progenitor cells (NPCs) were cultured in neural expansion medium (DMEM/F12 supplemented with 1× B27 and N2 [Life technologies], fibroblast growth factor-8a [FGF8a, 100 ng/ml, Life technologies], sonic hedgehog [SHH C25II, 200 ng/ml, Life technologies]; heparin [2 μg/ml, Life technologies]; 100 μM 2-mercaptoethanol [Life technologies]; 1× non-essential amino acids [NEAA, Life technologies] and ascorbic acid [200 μM, Sigma] for 5 days and finally in cortical neuronal differentiation medium (Neurobasal medium supplemented with L-glutamine [2 mM, Life technologies]. 1× non-essential amino acids, 1× B27 and N2 supplements) for 10, 15 and 20 days before further analysis.

### Transfection of iPSC-derived neurons

siRNAs were introduced by reverse transfection using RNAiMAX (Life Technologies) according to the manufacturer’s protocol. For both Mfn2 siRNA and scrambled siRNA controls transfected neurons, the cells were plated at 0.5 × 10^5^ neurons/well in 12-well plates and were transfected with siRNAs 12 days after incubated in neural differentiation medium. In this study, relatively high siRNA concentrations (40 nM) are needed to achieve efficient knockdown, which is sustained over many days even after culture medium change^35^. The medium was half changed with fresh neural differentiation medium every 48 hours. The cells were harvested for analysis 8 days (20 days in differentiation in total) after siRNAs transfection. The target siRNAs are Mfn2 siRNA (Dharmacon RNA Technologies, L-012961-00) and the non-targeting scrambled control siRNA (Dharmacon RNA Technologies, D-001810-10-05).

For Mfn2 overexpression, NPCs cultured in neural expansion medium for 5 days were transfected with yellow fluorescent protein plasmid Mfn2-YFP (Addgene plasmid 28010) or empty vector alone using Lipofectamine 2000 (Invitrogen, 11668019), according to manufacturer’s instructions. The medium was half changed with fresh neural differentiation medium every 48 hours. The cells were harvested for analysis 10 days (10 days in differentiation in total) after transfection.

The efficiency of Mfn2 knock-down or overexpression was validated by immunostaining and immunoblotting for Mfn2 (Figs 4A–C and 7A,B).

### Immunoblotting analysis

Differentiated cells cultured in neuronal differentiation medium for 10, 15 and 20 days were washed with ice-cold PBS and proteins extracted with 150 μl of lysis buffer. After centrifugation at 12,000 × g for 10 min at 4 °C, we collected the supernatant and determined protein concentrations; we boiled 30 μg proteins in protein loading buffer for 5 min, separated the proteins on a 10% SDS polyacrylamide gel, and subsequently transferred to nitrocellulose membranes. Nonspecific binding was blocked by the incubation in TBST (20 mM Tris-buffered saline with 0.1% Tween 20, pH 7.5) containing 5% nonfat dried milk for 1 hour at room temperature. Membranes were incubated with the following primary antibodies: rabbit anti-PAX6 (Paired box protein 6, 1:1000, 42–6600, Invitrogen), rabbit anti-Oct-4 (Octamer-binding transcription factor 4, 1:1000, Life Technologies), mouse anti-TuJ1 (class III β-tubulin, 1:10000, Sigma), rabbit anti-syn (synaptophysin, 1:5000, A0010, Dako), and rabbit anti-Mfn2 (1:1000, WH0009927M3, Sigma) overnight at 4 °C. After three washes with TBST, membranes were incubated for 2 h with horseradish (HRP)-conjugated secondary antibodies (Pierce Chemical Company, USA) and developed using enhanced chemiluminescence (ECL Amersham Biosciences, England). To ensure equal protein loading of the samples, the same membrane was probed with anti-mouse β-actin monoclonal antibody (Sigma Aldrich, MO) at a 1:10,000 dilution.

### Immunocytochemistry

The BM2-3 hiPSCs, less than 10 passages, the induced cortical neurons, after differentiation for 5, 10, 15 and 20 days, with different treatment, or the attached neural aggregates, all cultured on coverslips, were fixed with 4% ice-cold paraformaldehyde for 5 min and then permeabilized with PBS containing 0.1% Triton and 5% goat serum for 1 h followed by incubation with the following primary antibodies: rabbit anti-Oct4 (1:2500, A13998, Life Technologies) and mouse anti-Nanog (1:2500, ab173368, Abcam); rabbit anti-SOX2 (1:2500, A13992; Life Technologies) and rat anti-SSEA3 (1:500, 41–4400, Life Technologies); rabbit anti-PAX6 (1:1000, 42–6600, Invitrogen) and mouse anti-Ki67 (1:1000, 556003, BD Pharmingen™); or rabbit anti-Syn (1:2000, A0010, Dako), mouse anti-MAP2 (1:8000, sc-33796, Santa Cruz Biotechnology) and rabbit anti-Mfn2 (1:1000, WH0009927M3, Sigma) at 4 °C for 16 h. Cells were incubated with combination of Alexa Fluor 594-conjugated goat anti-rabbit IgG and 488 goat anti-mouse/rat IgG (1:1000, Invitrogen), Alexa Fluor 488-conjugated goat anti-rabbit IgG and 594 goat anti-mouse IgG (1: 1000, Invitrogen), or Alexa Fluor 594-conjugated goat anti-mouse IgG and 633 goat anti-rabbit IgG (1: 1000, Invitrogen) for 1 h at room temperature. After washing with PBS, neurons were covered with Vectashield mounting medium (H-1000, Vector Laboratories). Images were acquired (equal exposure for all groups) under confocal microscopy (Leica) and analyzed using the Universal Metamorph Image Program.

We measured synaptic density of cultured neurons by counting the number of synaptophysin-positive clusters in neuronal dendrites and puncta per 100 microns of dendrite (presented as the number of synaptophysin clusters per 100 microns of dendrite) and calculated by dividing the length of the dendrite as we previously described^36^.

### Real-time PCR measurement

RNA was extracted identified cells by using TRIzol reagents (Invitrogen, Carlsbad, CA, USA) according to the manufacturer’s protocol as described in our previous study^36^. Total RNA (1 μg) was used for the synthesis of cDNA with TaqMan Reverse Transcription Reagents kit (Roche Applied Biosystems). Real time-PCR was performed on an ABI Prism 7900 Sequence Detection System (Applied Biosystems) with TaqMan PCR Master Mix. Real-time PCR was utilized for quantification of gene expressions of *MAP2* (Hs00258900_m1, Applied Biosystems). The ribosomal RNA (18S) probes and primers came from Applied Biosystems (Mm03928990-g1, Applied Biosystems). Data are calculated using the 2 −ΔΔCt method as described by the manufacturer and are expressed as fold-increase over the indicated controls (1.0) in each figure.

### Electrophysiology analysis

Electrophysiologic experiments were performed on day-10, −15~16 and −20 differentiated cortical neurons. Electrophysiology recordings were performed as described previously^37^. We analyzed cells at indicated times after induction, recording resting membrane potential, spontaneous, and evoked action potentials using current clamp whole-cell configuration. Evoked action potentials were recorded at a holding potential on resting membrane potential; step currents ranging from −10 pA to +90 pA were injected at 20 pA increments to elicit action potentials. Whole-cell currents including sodium currents and potassium currents were recorded at a holding potential of −70 mV, with voltage steps ranging from −70 mV to +50 mV delivered at 10 mV increments. Spontaneous postsynaptic currents (sPSCs) were recorded at a holding potential of −70 mV with voltage clamp configuration. The pipette solution for patch-clamp experiments contained (in mM) 130 K-gluconate, 10 KCl, 5 MgCl_2_, 5 HEPES, 0.6 EGTA, 0.06 CaCl_2_, 2 MgATP, and 0.2 Na_2_GTP, pH adjusted to 7.2 with KOH. The recording bath solution contained (in mM) 119 NaCl, 5 KCl, 20 HEPES, 30 glucose, 2 CaCl_2_ and 2 MgCl_2_, pH adjusted to 7.3 with NaOH. We acquired whole-cell patch clamp recordings using a MultiClamp 700B amplifier, Digidata 1440A, and Clampex data acquisition software (Molecular Devices) at room temperature.

### Measurement of respiratory chain complex enzyme activities and ATP levels

Enzyme activities in complex I (NADH-ubiquinonereductase), complex IV (cytochrome c oxidase, CcO), and ATP levels were determined as described previously^8^.

NADH: ubiquinons oxidoreductase (COXI) enzyme activity was determined in 25 mM potassium buffer containing KCl, TrisHCl and EDTA (pH 7.4). The change in absorbance was monitored at 340 nm wave length every 20 seconds for 6 minutes using an Amersham Biosciences Ultrospect 3100 pro spectrophotometer.

In the presence of mitochondria (50 μg protein), 2 μg/ml antimycin, 5 mM magnesium chloride, 2 mM potassium cyanine and 65 μM coenzymes Q1 were added and the oxidation of NADH was recorded for 3 min; then 2 μg/ml rotenone was added and absorbance was measured for another 3 min. The reaction was triggered by the addition of 50 μl ferrocytochrome c substrate solution (0.22 mM) into the cuvette. The changes in absorbance of cytochrome c at 550 nm was recorded immediately using a kinetic program with 5 seconds delay, 10 seconds interval and total 6 readings on an Ultrospect 3100 Pro spectrophotometer. ATP levels were determined using an ATP Bioluminescence Assay Kit (Roche) following the manufacturer’s instruction^38^,^39^. Briefly, cells were harvested using the provided lysis buffer, incubated on ice for 15 minutes, and centrifuged at 13,000 g for 10 minutes. ATP levels were measured using a Luminescence plate reader (Molecular Devices) with an integration time of 10 seconds.

### Determination of TMRM and MitoSOX Red fluorescence staining intensities

Differentiated neuronal cells were seeded at low density onto Lab-Tek eight-well chamber slides (10,000 cells/well). Mitochondrial ROS generation was determined using MitoSOX Red (Invitrogen), a unique fluorogenic dye highly selective for detection of superoxide production in live cell mitochondria. Cells were incubated with fresh medium containing 2.5 μM MitoSOX for 30 min at 37 °C. As a fluorescence cationic indicator taken up into mitochondria in a potential-dependent manner, TMRM (100 nM; Invitrogen) were used in this study to evaluate the changes of mitochondrial membrane potential level. Fluorescent signals MitoSOX and TMRM were quantified from the same area of mitochondria indicated by MitoTracke Green (MTGreen, 100 nM, Invitrogen) co-staining for 30 min, using NIH Image J software. Fluorescence images were acquired on a Leica SP5 confocal microscope and analyzed using Leica LAS AF software (Leica Wetzlar). Excitation wavelengths were 543 nm for MitoSOX and TMRM and 488 nm for MTGreen, respectively. We used MetaMorph (Molecular Devices) and NIH Image J software for quantification and measurement of fluorescent signals of mitochondrial length and mitochondrial density. Mitochondrial size, shape, density, and fluorescent intensity were quantified by an investigator blinded to experimental groups. Mitochondria from 20–25 randomly selected cells were measured and quantified.

To evaluate intracellular ROS levels and to confirm the results obtained from MitoSOX red staining, election paramagnetic resonance (EPR) spectroscopy is also applied in this study, as described in Supplementary Information.

### Axonal mitochondrial trafficking recording and data analysis in differentiated neuronal cells

Axonal mitochondria were visualized following transfection with pDsRed2-mito (Clontech) in differentiated neuronal cells using lipofectamine LTX and plus regent (Invitrogen) according to the manufacturer’s protocol. Three to four days after transfection, time-lapse recordings of labeled mitochondrial movement were acquired on a Carl Zeiss (Axiovert 200) microscope with incubation system (PeCon) to maintain differentiated neuronal cells at 37 °C during image collection. Collection of image stacks and velocity measurements were made using the AxioVision Software as previously described^39^,^40^. For standard recordings, images of mitochondria in one process per differentiated neuronal cell were collected every 3 s for 2 min. Only the proximal segment of the axon was acquired and recorded.

Mitochondria in each frame of every video recording were individually tracked using AxioVision Software and the average velocity was calculated during the 2-min recording period. The average velocity of every mitochondrion in one measured process in each cell was then averaged to obtain the average velocity for mitochondrial movement per process. In addition, the percentage of movable mitochondria, mitochondrial total traveling distance (total distance traveled irrespective of direction during the recording period), single mitochondrial length and mitochondrial density in each process were determined according to previous studies with modifications^8^,^41^. Exposure periods (30–50 ms) were kept at a minimum to limit phototoxicity.

### Statistical analysis

Data are presented as mean ± SEM. Statistical analysis was performed using Statview software (SAS Institute, Version 5.0.1). One-way ANOVA was used for repeated measure analysis, followed by Fisher’s protected least significant difference for post hoc comparisons. P < 0.05 was considered significant.

## Additional Information

**How to cite this article**: Fang, D. *et al*. Mfn2 is Required for Mitochondrial Development and Synapse Formation in Human Induced Pluripotent Stem Cells/hiPSC Derived Cortical Neurons. *Sci. Rep.* **6**, 31462; doi: 10.1038/srep31462 (2016).

## Supplementary Material

Supplementary Information
