# Supplementary material for: Mfn2 is Required for Mitochondrial Development and Synapse Formation in Human Induced Pluripotent Stem Cells/hiPSC Derived Cortical Neurons
Source: Sci Rep. 2016 Aug 18;6:31462. doi: 10.1038/srep31462 (PMC4989148; doi:10.1038/srep31462)
Supplement: Supplementary Information [file srep31462-s1.pdf]

Mfn2 is Required for Mitochondrial Development and Synapse Formation in Human Induced Pluripotent Stem Cells/hiPSC Derived Cortical Neurons

Du Fang [1,3], Shijun Yan [1,3], Qing Yu [1,2], Doris Chen [1], and Shirley ShiDu Yan [1]*

[1]*Department of Pharmacology and Toxicology, and Higuchi Bioscience Center, School of Pharmacy, University of Kansas, Lawrence, KS66047*

[2]*State Key Laboratory of Oral Diseases, West China Hospital of Stomatology, Sichuan University, Cheng Du, China*

[3]*These authors contributed equally to this work*

*Corresponding author: Dr. Shirley ShiDu Yan,

Departments of Pharmacology and Toxicology and Higuchi Bioscience Center, School of Pharmacy, University of Kansas, and 2099 Constant Ave.

Lawrence, KS 66047, USA

E-mail: shidu@ku.edu,

Tel: 1-785-864-3637

Running Title: **Mfn2 in hiPSC derived cortical neurons**

Supplementary Information

hiPSC characterization

We assessed pluripotency in BM2-3 hiPSCs. These cells were immunoreactive for pluripotency markers: Nanog (**Supplementary Figure S1AI**), OCT4 (**Supplementary Figure S1AII**), SSEA3 (**Supplementary Figure S1AV**) and Sox2 (**Supplementary Figure S1AVI**). Our iPSCs were successfully kept in an undifferentiated state for more than 17 passages. The cells (17 passages) formed uniform-sized embryoid bodies (EBs) 24 hours after being plated into AggreWell™800 plates (**Supplementary Figure S1B**). 1-2 days after attachment, prominent neural rosette structures appeared in neural aggregates. About 98% of attached neural aggregates had polarized rosettes covering >50% of their area (**Supplementary Figure S1C**). Rosettes were formed by cells expressing proteins characteristic of progenitor markers of PAX6 (**Supplementary Figure S1C**).

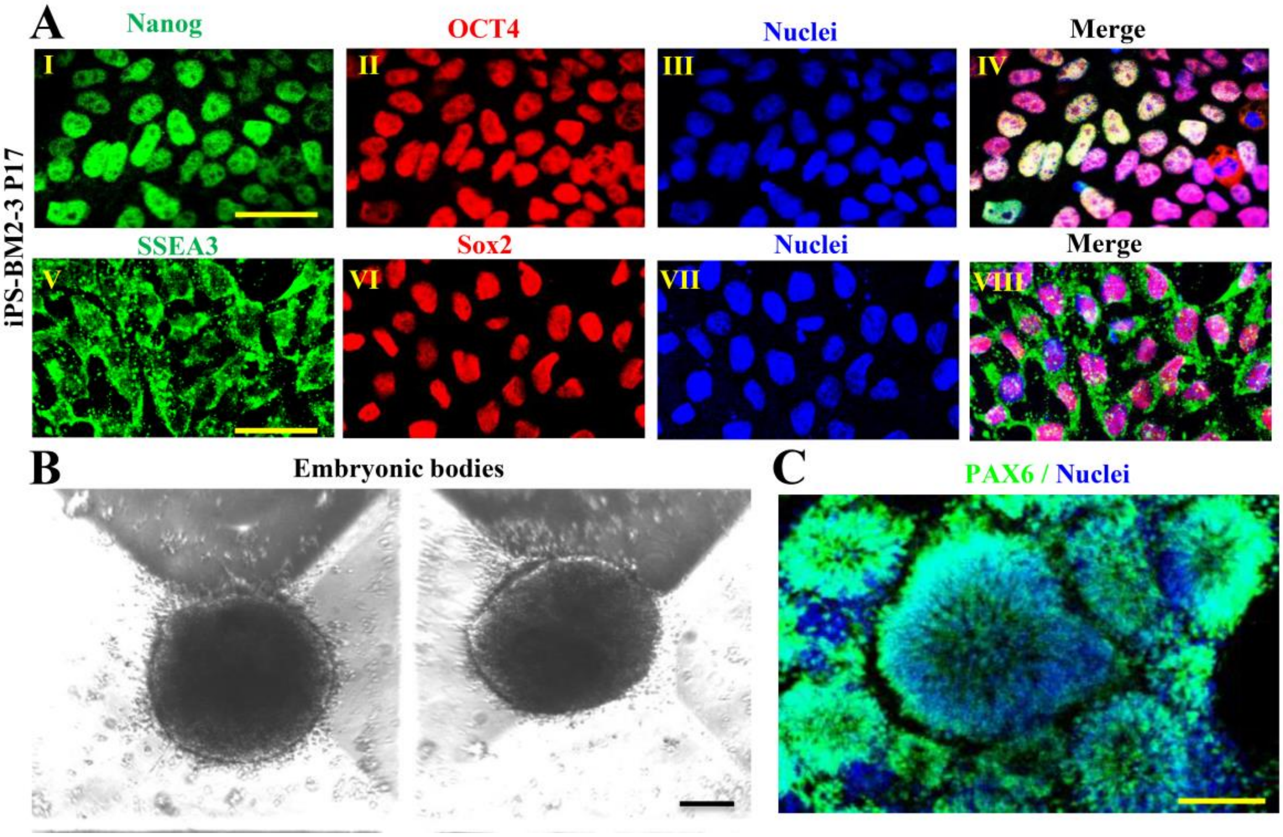

Supplementary Figure S1. Characterization and identification of hiPSC line derived from normal human bone marrow fibroblasts. (**A**) Normal human bone marrow fibroblast-derived hiPSCs. Bone Marrow Clone 2-3(BM2-3) was selected for analysis. hiPSC-BM2-3 cells expressed pluripotency markers: NANOG (**AI**), OCT4 (**AII**), SSEA3 (**AV**) and Sox2 (**AVI**). (**B** and **C**) Formation of embryonic bodies (EB, **B**), neural aggregates and rosettes (**C**) of hiPSC-BM2-3 cells. Images in **B** show EB formation after forced aggregation of hiPSC-BM2-3 cells using aggrewell plates. Image in **C** shows rosette formations with rosette-like structures co-expressing early-stage precursor cells marker PAX6 (Green). Nuclei were stained by DRAQ5, a far-red emitting fluorescent DNA dye, for 10 min at room temperature. Scale bars = 50 μm.

Screen of TUJ1, synaptophysin /Syn and Mfn2 expression levels in hiPSC induced cortical neurons differentiated for 20 to 30 days

We investigated the protein levels of Mfn2, TUJ1 and Syn in cortical neurons derived from hiPSCs for 20 to 30 days (**Supplementary Figures S2A-D**). The expression levels of TUJ1, Syn and Mfn2 reached peak expression levels after 20 days of differentiation.

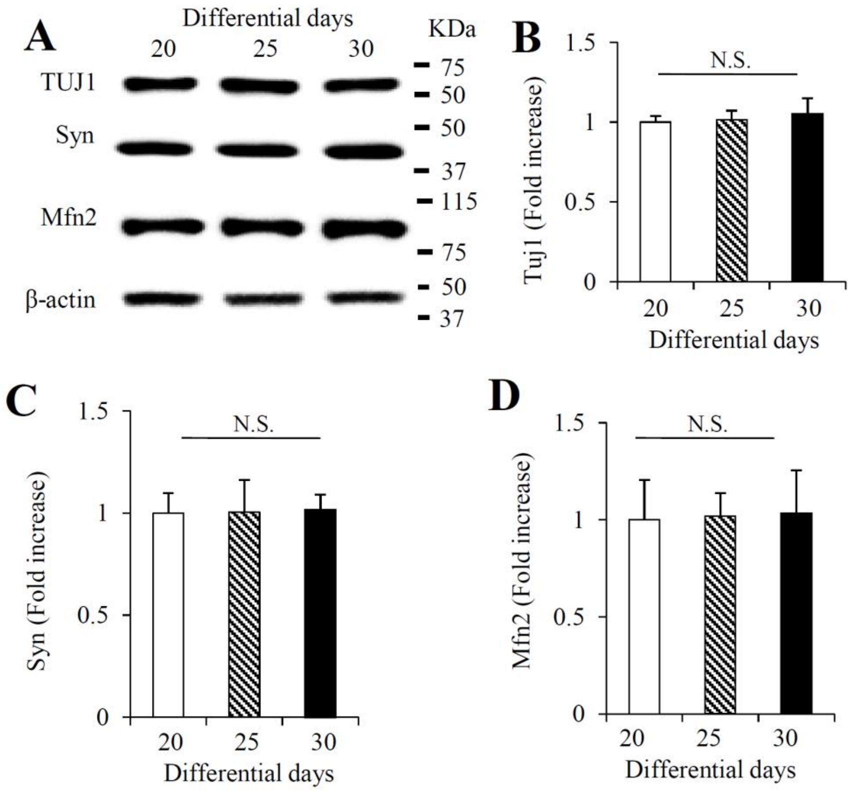

Supplementary Figure S2. Expression levels of TUJ1, Syn and Mfn2 in the hiPSC line-induced human cortical neurons differentiated for different days. (**A-D**) Immunoblotting analysis for TUJ1, Syn and Mfn2 proteins expression levels in the hiPSC line-induced human cortical neurons differentiated for 20, 25, and 30 days. Representative immunoblots for TUJ1, Syn, Mfn2 and β-actin (**A**). Quantifications of TUJ1 (**B**), Syn (**C**) and Mfn2 (**D**) expression levels are normalized to β-actin using NIH image J program (n = 3).

Effect of Mfn2 on ROS levels in hiPSCs differentiated cortical neurons

Besides MitoSOX staining, we also analyzed ROS levels in hiPSCs differentiated cortical neurons using EPR method. Consistent with the results from MitoSOX staining (**Figures 3H-I**), our EPR results also demonstrated increased ROS levels in hiPSCs induced neurons during differentiation (**Supplementary Figures S3A-B**). Mfn2 knockdown decreased ROS levels in day 20 of differentiated neurons (**Supplementary Figures S3C-D**), while its overexpression increased ROS levels in day 10 of differentiated neurons (**Supplementary Figures S3E-F**).

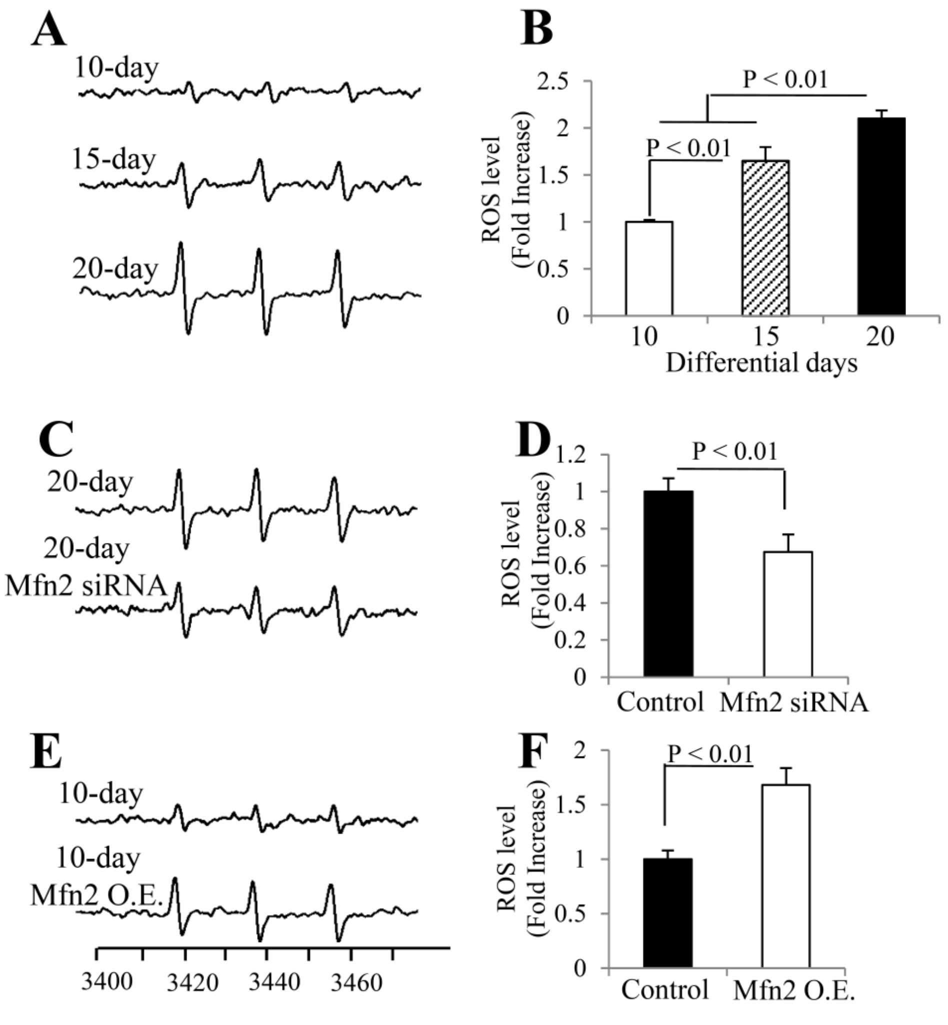

Supplementary Figure S3. Effect of Mfn2 on ROS levels of in the hiPSC line-induced human cortical neurons differentiated for different days. EPR analysis on hiPSC-derived neurons differentiated for 10, 15 and 20 days (**A-B**), day 20 neurons with Mfn2 knockdown (**C-D**) and 10 neurons with Mfn2 overexpression (**E-F**). Quantification of EPR (**B, D** and **F**) spectra and representative spectra of EPR (**A, C** and **E**) in the indicated groups of neurons.

Supplementary methods

Evaluation of intracellular ROS levels

Evaluation of intracellular ROS levels was accessed by election paramagnetic resonance (EPR) spectroscopy as described in our previous study[1,2]. CMH (cyclic hydroxylamine 1-hydroxy-3-methoxycarbonyl-2, 2, 5, 5-tetramethyl-pyrrolidine, 100μM) was incubated with cultured cells for 30 min, and then washed with cold PBS. The cells were collected and homogenized with 100μl of PBS for EPR measurement. The EPR spectra were collected, stored, and analyzed with a Bruker EleXsys 540 x-band EPR spectrometer (Billerica, MA) using the Bruker Software Xepr (Billerica, MA).

Reference

1 Fang, D. *et al.* Increased neuronal PreP activity reduces Abeta accumulation, attenuates neuroinflammation and improves mitochondrial and synaptic function in Alzheimer disease's mouse model. *Human molecular genetics* **24**, 5198-5210, doi:10.1093/hmg/ddv241 (2015).

2 Fang, D. *et al.* Increased Electron Paramagnetic Resonance Signal Correlates with Mitochondrial Dysfunction and Oxidative Stress in an Alzheimer's disease Mouse Brain. *Journal of Alzheimer's disease : JAD* **51**, 571-580, doi:10.3233/JAD-150917 (2016).
